# Retraction Note: Molecular and therapeutic effect of CRISPR in treating cancer

**DOI:** 10.1007/s12032-025-02692-7

**Published:** 2025-03-24

**Authors:** Sawani Rodrigo, Kaveesha Senasinghe, Sameer Quazi

**Affiliations:** 1 Human Genetics Unit, Faculty of Medicine, University of Colombo, Colombo, Sri Lanka; 2 GenLab Biosolutions Private Limited, Bengaluru, Karnataka 560043 India; 3 Department of Biomedical Sciences, School of Life Sciences, Anglia Ruskin University, Cambridge, UK; 4 School of Health Sciences, The University of Manchester, Manchester, UK; 5 SCAMT Institute, ITMO University, St. Petersburg, Russia

**Retraction Note: Medical Oncology (2023) 40:81** 10.1007/s12032-022-01930-6

The Editor-in-Chief has retracted this article following an institutional investigation by the University of Manchester. After analysing the findings of this investigation, the Publisher reassessed the editorial handling and peer review of this publication and found evidence to suggest that latter was compromised. The Editor-in-Chief therefore no longer has confidence in the integrity of this review article.

Sawani Rodrigo and Kaveesha Senasinghe disagree with this retraction. Sameer Quazi has not replied to correspondence from the Publisher.

